# A Narrative Review on the Effectiveness of Bone Regeneration Procedures with OsteoBiol^®^ Collagenated Porcine Grafts: The Translational Research Experience over 20 Years

**DOI:** 10.3390/jfb13030121

**Published:** 2022-08-18

**Authors:** Tea Romasco, Margherita Tumedei, Francesco Inchingolo, Pamela Pignatelli, Lorenzo Montesani, Giovanna Iezzi, Morena Petrini, Adriano Piattelli, Natalia Di Pietro

**Affiliations:** 1 Department of Medical, Oral and Biotechnological Sciences, “G. d’Annunzio” University of Chieti-Pescara, 66013 Chieti, Italy; 2 Center for Advanced Studies and Technology-CAST, “G. d’Annunzio” University of Chieti-Pescara, 66013 Chieti, Italy; 3 Department of Medical, Surgical, and Dental Sciences, University of Milan, 20122 Milan, Italy; 4 Department of Interdisciplinary Medicine, University of Bari “Aldo Moro”, 70124 Bari, Italy; 5 Department of Oral and Maxillofacial Sciences, “Sapienza” University of Rome, 00131 Rome, Italy; 6 Private Practice, 00187 Rome, Italy; 7 School of Dentistry, Saint Camillus International University of Health and Medical Sciences, Via di Sant’Alessandro 8, 00131 Rome, Italy; 8 Fondazione Villa Serena per la Ricerca, 65013 Città Sant’Angelo, Italy; 9 Casa di Cura Villa Serena del Dott. L. Petruzzi, 65013 Città Sant’Angelo, Italy

**Keywords:** bone regeneration, bone defects, maxillary defects, oral surgery, xenografts, porcine bone grafts, biomaterials

## Abstract

Over the years, several bone regeneration procedures have been proposed using natural (autografts, allografts, and xenografts) and synthetic (i.e., metals, ceramics, and polymers) bone grafts. In particular, numerous *in vitro* and human and animal *in vivo* studies have been focused on the discovery of innovative and suitable biomaterials for oral and maxillofacial applications in the treatment of severely atrophied jaws. On this basis, the main objective of the present narrative review was to investigate the efficacy of innovative collagenated porcine bone grafts (OsteoBiol^®^, Tecnoss^®^, Giaveno, Italy), designed to be as similar as possible to the autologous bone, in several bone regeneration procedures. The scientific publications were screened by means of electronic databases, such as PubMed, Scopus, and Embase, finally selecting only papers that dealt with bone substitutes and scaffolds for bone and soft tissue regeneration. A total of 201 papers have been detected, including *in vitro*, *in vivo*, and clinical studies. The effectiveness of over 20 years of translational research demonstrated that these specific porcine bone substitutes are safe and able to improve the biological response and the predictability of the regenerative protocols for the treatment of alveolar and maxillofacial defects.

## 1. Introduction

Bone regeneration procedures are surgical techniques developed to restore the jaw defects provoked by tissue damage, infections, tooth loss, neoplasms, or local trauma [1,2,3]. Many different protocols have been adopted in accordance with the defect type (horizontal/vertical augmentation) [4,5,6,7], the local anatomy (anterior/posterior region of maxilla/mandibula) [8,9,10,11], the defect extension, and the planned rehabilitation [4,12,13,14]. The rationale of these procedures is to obtain a durable regeneration of the hard/soft tissue interface after the organization of a blood clot, which promotes the local new bone formation [15,16,17]. The use of xenografts and alloplastic bone substitutes represents a useful and safe technique that takes advantage of the high manageability of these products, avoiding the need for a donor site for autologous graft retrieving [17,18,19]. The effectiveness of these products has been evaluated by different studies conducted in various research centers around the world. These studies have been developed on a progressive scale, starting from *in vitro* studies on cell cultures, proceeding with *in vivo* studies on animal models, and finally with human studies, which allow for strengthening the 20-year work experience in translational research activity. In particular, both the histological and histomorphometric investigations performed at the microscopic level are able to reveal the bone response to the graft, providing strong knowledge about the bone scaffold behavior, the resorption process, the local bone neoformation, and the long-term persistent response of the regenerated tissues. Moreover, these methodologies have been associated with other techniques, such as Scanning Electron Microscopy (SEM), Transmission Electron Microscopy (TEM), Atomic Force Microscopy (AFM) and Synchrotron Micro-CT, in order to improve the biomaterial surface characterization and information about the physicochemical and biological compositions. This methodological approach steers clinicians towards the correct choice of the scaffold shape (i.e., particulate/block), the surgical procedure, and the graft manipulation and stabilization techniques, in order to increase the predictability of the procedure. In these terms, the aim of the present review was to describe the effectiveness of several protocols for the alveolar/maxillofacial bone and soft tissue regeneration using different OsteoBiol^®^ innovative collagenated porcine bone grafts.

## 2. Materials and Methods

The screening of the studies was performed using the electronic databases PubMed, Scopus, and Embase, through the research of specific keywords: Piattelli A AND porcine bone biomaterials; Piattelli A AND porcine bone biomaterials AND jawbone regeneration; Piattelli A AND porcine granules; Piattelli A AND porcine bone blocks; Piattelli A AND porcine collagen bone barriers; Piattelli A AND porcine collagen membranes; OsteoBiol^®^ AND porcine bone biomaterial; OsteoBiol^®^ AND jawbone regeneration; OsteoBiol^®^ AND maxillofacial regeneration; OsteoBiol^®^ AND porcine granules; OsteoBiol^®^ AND porcine bone blocks; OsteoBiol^®^ AND porcine collagen bone barriers; OsteoBiol^®^ AND porcine collagen membranes.

The manuscripts were then evaluated through a qualitative synthesis.

### 2.1. Inclusion Criteria

The studies published up to January 2021 were evaluated with no language restrictions. The identified studies were limited to papers that dealt with collagenated porcine bone substitutes and scaffolds for bone and soft tissue regeneration during the last 20 years. No restrictions about the use of barrier membranes were applied to the systematic research process. The inclusion criteria considered human studies, *in vitro* research and reports, and animal model investigations. The off-topic publications were excluded from the investigation. The articles were then classified in accordance with the surgical procedure and the study design.

### 2.2. Selection of the Studies

The screening of the study data and papers was performed independently by two calibrated and expert reviewers (M.T. and A.P.). After a first check, all the abstracts of the identified papers were evaluated as the 1st level of screening. The reviews and book chapters were excluded from the qualitative analysis. A description of the reasons for exclusion was drafted, concerning not considered articles. The full text of the included papers was obtained, and then, they were classified for the qualitative synthesis. For this purpose, a specially designed data form was used (Excel Office Microsoft, Redmond, WA, USA).

**Figure 1:**
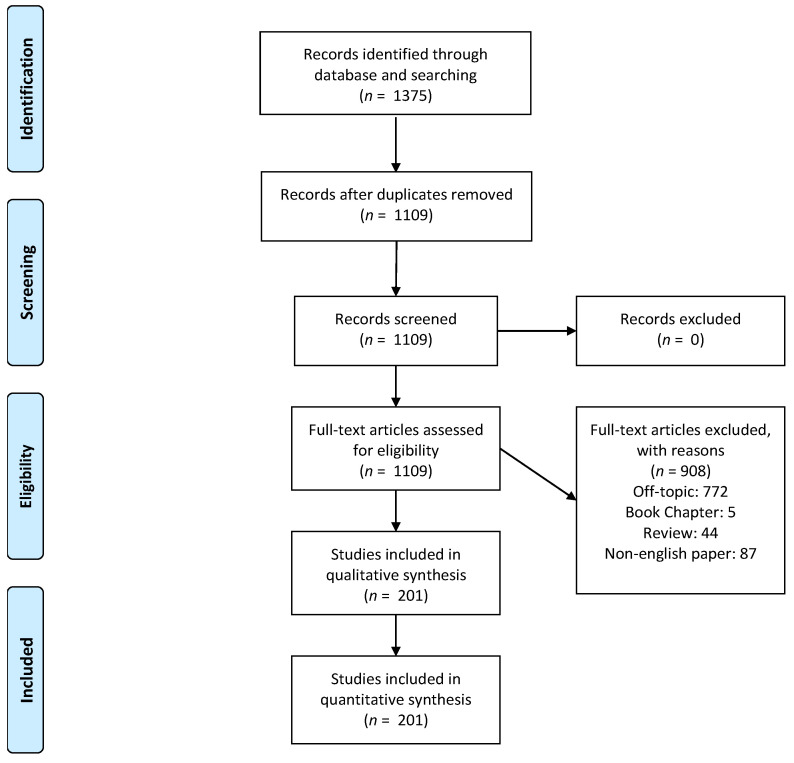
PRISMA Flowchart of the study design and manuscript-selection process.

A total of 1375 manuscripts have been detected by the electronic database research. A total of 266 duplicates have been removed, and 1109 papers have been considered for the full-text eligibility evaluation. A total of 44 literature reviews, five book chapters, 87 papers written in non-English grammar, and 772 off-topic manuscripts were excluded. In the end, a total of 201 papers have been included in the final analytical synthesis (Figure 1).

### 2.3. Description of the Porcine Grafts

**Figure 2:**
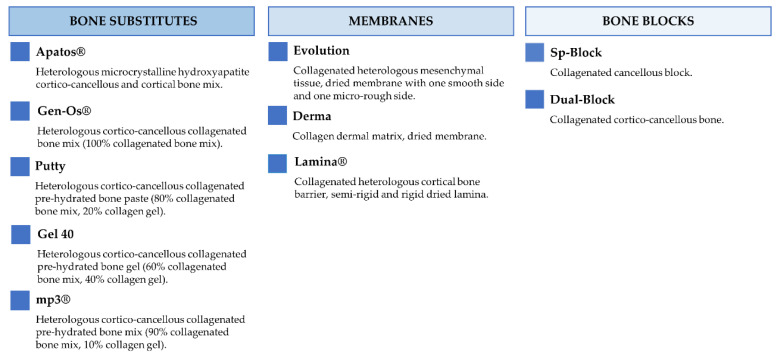
Description of the characteristics regarding OsteoBiol^®^ products.

**Figure 3:**
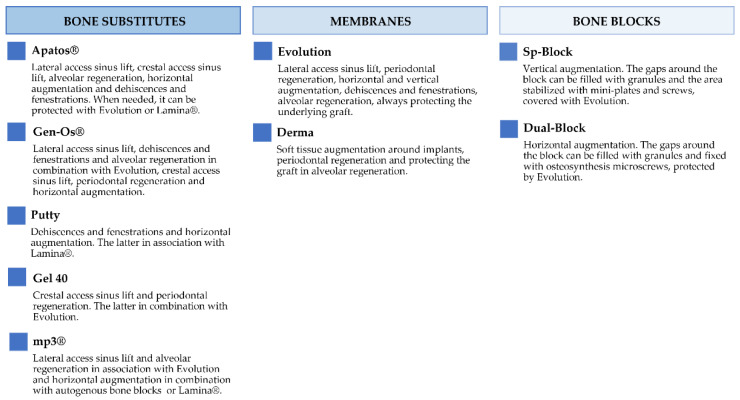
Description of the clinical applications of OsteoBiol^®^ products.

Figure 2 and Figure 3 report the characteristics and the clinical applications of the different biomaterials (OsteoBiol^®^, Tecnoss^®^, Giaveno, Italy) used and cited in the selected papers. All of them are porcine collagenated xenografts and show high biocompatibility and osteoconductive properties [20,21]. A dedicated product has been developed for every clinical indication, trying to provide the best handling, granulometry, and consistency, in order to achieve ideal regenerative results [22]. In particular, the dual-phase heterologous bone matrix granules are composed of a mineral phase and a xenogenic collagen phase, which is able to provide the best biocompatibility, a chemical composition similar to autogenous bone, gradual resorption of the bone matrix with the replacement by the newly formed bone at re-entry time, and a high angiogenic potential [23,24,25,26]. These elements are critical for a successful bone regeneration procedure that sometimes can be further improved with the association of some of these xenografts.

## 3. Results

The main effective results for each biomaterial used alone or in combination have been schematically divided and summarized in the tables below [Table 1, Table 2, Table 3, Table 4, Table 5, Table 6, Table 7 and Table 8], on the basis of the clinical indication they have been specifically designed for.

In summary, all the *in vitro*, experimental, and clinical results described in Table 1, Table 2, Table 3, Table 4, Table 5, Table 6, Table 7 and Table 8 suggested that, during the last 20 years, the OsteoBiol^®^ collagenated biomaterials have shown reliable outcomes in terms of biocompatibility, morbidity, new bone formation, and bone and soft tissue regeneration, according to expert surgeons’ experience.

## 4. Discussion

The number of studies reporting surgical techniques for bone regeneration and the clinical effectiveness of bone substitutes and xenografts has greatly increased over the last years, with high predictability and stability of the regenerated alveolar bone ridges [9,18,219]. The treatment of bone defects represents a clinical occurrence that requires optimal management of the three-dimensional stability of the grafts and regenerative spaces. In this way, blood-clot stability plays a key role in new bone formation and the morphological restoration of the atrophied bone ridge [220].

The effectiveness of graft implantation is affected by a biunivocal biological relationship between the host tissue and the bone substitutes that has been investigated by numerous histological studies on retrieved biopsies [221].

In many *ex vivo* studies conducted by using porcine graft specimens, the histologic and histomorphometric evaluations reported newly formed bone in contact with the scaffolds and an evident presence of cells in the osteocyte lacunae [7,24,25,27,222].

This evidence has been corroborated by the clinical success of these biomaterials, which confirmed the histologic and histomorphometric findings and showed an intimate apposition of newly formed bone in contact with the porous porcine-derived biomaterials, especially in maxillary sinus augmentation procedures [28,85,89,90,93,97,99,100,101,110,111,116,117,119].

In addition, the results obtained from *ex vivo* and clinical data have been supported by *in vitro* studies, which demonstrated the osteoblast differentiation and bone regeneration capabilities together with the angiogenic potential of the OsteoBiol^®^ bone matrix [21,23,26,178,183,184,185,194,197].

With reference to graft resorption, many studies revealed the nearly complete substitution of membranes and the ongoing resorption of collagenated bone particles within 6 months. Especially, Wachtel et al. [123] reported that the biodegradation of the cortical bone Lamina^®^ was almost complete after 6 months, with varying degrees of residual graft particles. Cardaropoli et al. [30] confirmed the presence of a marginal residual graft rate (24.5%) of Gen-Os^®^ biomaterial, covered by Evolution^®^ collagen membrane to preserve the bone socket, just after 4 months from implant insertion. Additionally, another clinical study [95] reported a high resorption rate of mp3^®^, with 13.55% of residual grafting material after 5 months, that reached 12.3% within 12 months [24]. Considering that the limit for the residual volume of bone grafts for successful implant placement is set at 40% [223], these values are considerably lower.

Regarding the aforementioned residual graft limit, it should be considered that only Apatos Cortical^®^ showed a higher residue percentage (around 30%) after many years from the surgery, although it stayed within 40%, comparable to the different types of xenografts present in the market [96,224].

However, these histological findings allow for adequate preservation of the grafted volume and do not appear to negatively affect the predictability of regenerative procedures and the survival rate of the dental implant in regenerated sites [225].

Overall, based on the data discussed, it appears clear that, due to the unique properties of these xenografts, an adequate preservation of graft volume and an improved new bone formation have been achieved.

In addition, the literature proved that OsteoBiol^®^ materials could be used alone or in combination both for the regeneration of bone defects and soft tissue augmentation. For example, in the latter case, membranes, such as Derma, can be used alone as an alternative to connective tissue graft to improve the quality of keratinized tissues [166,171,172,173,174]. Apatos^®^, instead, is a universal filler that can be employed to treat peri-implant defects and two-wall defects [68,74]. Moreover, thanks to its granulometry, Apatos^®^ fits well in big sockets, e.g., after molar extractions [41]. For this reason, sinus lift procedures (with crestal or lateral access) [85,91] can be performed with Apatos^®^ as a bone substitute, as well as surgeries for horizontal regenerations. Finally, as an example of a combination of materials, Apatos^®^ grafts can be protected with Evolution membrane [59] to reach a better ridge preservation compared to non-preserved size.

Although the effectiveness of using these biomaterials has been summarized in the results (Table 1, Table 2, Table 3, Table 4, Table 5, Table 6, Table 7 and Table 8) and discussed in this section, it is necessary to recognize that this narrative review has potential weaknesses. The main limitations include: (i) the manuscript does not contain all the reports in the field of “effectiveness of bone regeneration procedures with collagenated porcine grafts”, but only some selected publications that concern OsteoBiol^®^ biomaterials; (ii) the collected articles come from studies not only conducted by the authors of this review, but also by several other authors; (iii) the manuscript describes the individual works but does not quantify the results, and no statistical analysis is performed here; (iv) the manuscript does not compare the effectiveness of OsteoBiol^®^ products with other competitors, which are also successfully used for bone and soft tissue regeneration within the craniofacial area. However, our main goal was to summarize the achievements of these specific materials over the years.

Despite these limitations, we can conclude that the 20-year translational research experience showed the safety of these specific porcine bone substitutes and demonstrated their capability to improve the biological response and predictability of regenerative protocols for the treatment of alveolar and maxillofacial defects. For future perspectives, it will certainly be useful to extend the number of included studies, analyze and compare the success rate of each product, and perform longer-term histological and histomorphometric studies in order to better understand the resorption times of all these biomaterials. In this way, a systematic review could be performed to better highlight the advantages of using OsteoBiol^®^ collagenated porcine bone grafts with respect to other porcine substitutes.

## Figures and Tables

**Table 1:** Bone regeneration procedures with collagenated porcine xenografts: Alveolar Regeneration (ALR) and Alveolar Regeneration/Dehiscences and Fenestrations (ALR/DEH).

Reference	Clinical Indication	Biomaterial	Results
Covani U., 2004 [27]	ALR	Putty, Evolution	New bone formation after 4 months
Arcuri C., 2005 [28]	ALR	Putty	Grafting material completely substituted by trabecular bone tissue after 3 months
Barone A., 2008 [29]	ALR	mp3^®^, Evolution	High percentage of trabecular bone and mineralized tissue in ridge preservation after 7 months
Cardaropoli D., 2008 [30]	ALR	Gen-Os^®^, Evolution	85% preservation of initial ridge dimension; new bone formation; 25% residual graft particles
Crespi R., 2009 [31]	ALR	Gen-Os^®^	100% implant survival for implants placed in sockets grafted with MHA, CS, and PB (24-month follow-up)
Rossi R., 2010 [32]	ALR	Gen-Os^®^	Minimally invasive approaches (3D navigation systems) with immediate loading allow the development, maintenance, and stability of soft and hard tissue
Crespi R., 2011 [33]	ALR	Gen-Os^®^	Good biocompatibility and high osteoconductivity in alveolar bone grafting
Festa V.M., 2013 [34]	ALR	Gen-Os^®^, Lamina	Reduced hard tissue reabsorption after tooth extraction compared to EXT after 6 months
Barone A., 2012 [35]	ALR	mp3^®^, Evolution	Grafted sites allowed the placement of larger implants and required fewer augmentation procedures at implant placement (3-year follow-up)
Barone A., 2013 [36]	ALR	mp3^®^, Evolution	Grafted sites allowed the placement of longer or wider implants (4-month follow-up)
Barone A., 2015 [37]	ALR	mp3^®^, Evolution	No significant differences between flap and flapless techniques for tooth extraction and socket grafting procedures
Barone A., 2015 [38]	ALR	mp3^®^, Evolution	Bone levels improvement in mesial and distal sites by using xenograft and PRF
Lorenzon G., 2015 [39]	ALR	Gel 40	Good bone regeneration after 11 and 18 months from the implant placement
Thalmair T., 2013 [40]	ALR	mp3^®^	Covering the extraction socket with the free gingival graft allowed the maintenance of soft tissue volume, and minimized the buccal contour shrinkage
Barone A., 2016 [41]	ALR	Apatos^®^, mp3^®^, Evolution	After 3 months, there was less volume loss and ridge surface and a significantly smaller shrinkage of the basal area
Felice P., 2016 [42]	ALR	mp3^®^, Evolution	More failures and complications but better aesthetics results in immediate and immediate-delayed placed implants; similar bone level changes (4 months post-loading)
Barone A., 2016 [43]	ALR	mp3^®^, Evolution	Immediate implant procedures are a successful treatment when strict selection criteria and important surgical expertise are applied (3-year follow-up)
Barone A., 2017 [44]	ALR	mp3^®^, Apatos^®^, Evolution	The ridge preservation procedures showed better results compared to natural healing: no differences in maintenance of bone width between the biomaterials, but bone height better preserved with the cortical porcine bone
Alfonsi F., 2017 [45]	ALR	Gen-Os^®^, mp3^®^, Apatos^®^, Evolution, Lamina^®^	The cortico-cancellous porcine bone presented osteoconductivity, volume maintenance, new bone formation, and reabsorption of the xenograft without inflammation
Esposito M., 2017 [46]	ALR	mp3^®^, Evolution	More frequent failures but better aesthetics results at immediate and immediate-delayed placed implants; similar bone level changes (one-year post loading)
Scarano A., 2017 [47]	ALR	Gen-Os^®^, Evolution	Greater stability after the implant placement at the time of mandibular molar extraction
Barone A., 2017 [48]	ALR	Apatos^®^, mp3^®^	Reduced bone loss in both test groups when compared to naturally healing sockets; no preservation of the alveolar crest; 30% reduction in the estimates after healing (4-month analysis)
Giuliani A., 2018 [24]	ALR	mp3^®^, Evolution	Thinner and a greater number of trabeculae in the grafted sites; defects homogenously filled; improved strength of the socket; resorbed biomaterial and new bone formation over time; mp3 preserved and healed defects
Checchi V., 2017 [22]	ALR	Gen-Os^®^, Evolution	Immediate placement of wide diameter implants provided inferior aesthetic outcomes and delayed placement of normal-diameter implants (one-year post loading)
Crespi R., 2011 [49]	ALR	Apatos^®^	Absence of inflammation; bone formation in all treated sites; presence of biomaterial particles and connective tissue; same bone formation and resorption processes for the two biomaterials
Corbella S., 2017 [50]	ALR	Gen-Os^®^, mp3^®^, Apatos^®^, Evolution	No difference in bone formation between the biomaterials; calcium sulphate and beta-tricalcium phosphate faster resorbed; xenografts were less resorbable; allografts did not show higher bone formation than control; lower new bone formation with bovine bone than spontaneous healing; porcine bone and magnesium-enriched hydroxyapatite showed a higher new bone volume
Kilinc A., 2017 [51]	ALR	Evolution	The secondary closure was strongly favorable over the primary closure in terms of swelling and mouth opening; collagen membrane may support primary healing in terms of wound healing
Troiano G., 2017 [52]	ALR	mp3^®^, Gen-Os^®^, Apatos^®^, Lamina^®^, Evolution	Using bone graft covered by a resorbable membrane decreased alveolar ridge horizontal and vertical resorption after tooth extraction
Rossi R., 2017 [53]	ALR	mp3^®^, Evolution	Software technology by means of implant navigation systems allowed the achievement of optimal aesthetic and functional results
Scarano A., 2018 [54]	ALR	Apatos^®^, Evolution	Patients who developed implant displacement into the mandibular corpus must remove implants as soon as possible, as the bone healing does not allow the removal later
Nakajima Y., 2018 [55]	ALR	Gen-Os^®^, Evolution	More apical position of the coronal level of osseointegration with the presence of alveolar mucosa at implants
Chandrasekaran B., 2017 [56]	ALR	Gen-Os^®^	Synergistic use of PRF with bone grafts accelerated the healing process and ensured adequate bone filling
Barone A., 2014 [57]	ALR	mp3^®^, Evolution	The flapped procedure gave more negative results (increased resorption in width of the post-extraction site, less vertical bone resorption on the buccal aspect); the flapless procedure allowed the augmentation of the keratinized gingival width, soft tissue preservation, and improvement
Kivovics M., 2017 [58]	ALR	Gen-Os^®^, Evolution	Successful maintenance of the vertical and horizontal dimensions of the ridge; sufficient bone volume for implant placement in all sites (6 months after surgery)
Marconcini S., 2018 [59]	ALR	mp3^®^, Apatos^®^, Evolution	After 4 years, better ridge preservation (preserving marginal bone and achieving better aesthetic results around implants); the cortical porcine bone showed better clinical outcomes
Ramanauskaite A., 2019 [60]	ALR	Gen-Os^®^, mp3^®^, Apatos^®^, Derma, Evolution	Higher survival rates and lower marginal-bone-level loss for implants inserted into the previously grafted sockets
Faria-Almeida R., 2019 [61]	ALR	mp3^®^, Evolution	The use of membrane achieved better results
Felice P., 2020 [62]	ALR	mp3^®^, Evolution	No significant difference in failure, complications, or patient satisfaction between all the procedures, but more failures in immediate and early implants; smaller bone loss with immediate implants; better aesthetic results with immediate and early implants (3 years post-loading)
Felice P., 2020 [63]	ALR	mp3^®^	Outcomes were similar between the two groups in the presence of adequate bone volumes; peri-implant marginal bone loss was minimal in both groups (3 years post-loading)
Esposito M., 2021 [64]	ALR	Gen-Os^®^, Evolution	Ridge preservation and delayed placement of conventional 4- or 5-mm diameter implants showed better results (5 years post-loading)
Th Elaskary A., 2021 [65]	ALR	Lamina^®^	Optimum radiographic, aesthetic, and periodontal outcomes; minimized treatment time and number of surgical interventions; the protocol delimited infection and prepared sockets for implant placement (one-year after placement)
Tallarico M., 2016 [66]	ALR/DEH	Gen-Os^®^, Derma	High implant and prosthetic survival and success rates; good aesthetic outcomes (6 months post-loading)
Tallarico M., 2017 [67]	ALR/DEH	Gen-Os^®^, Derma	Both procedures showed successful results but waiting 4 months after tooth extraction and socket preservation procedures showed less marginal bone loss and a better aesthetic outcome (one-year post-loading)

**Table 2:** Bone regeneration procedures with collagenated porcine xenografts: Dehiscences and Fenestrations (DEH) and Dehiscences and Fenestrations/Lateral Access Sinus Lift (DEH/LASL).

Reference	Clinical Indication	Biomaterial	Results
Barone A., 2006 [68]	DEH	Putty, Apatos^®^, Evolution	Immediate implants and regenerative procedures to treat peri-implant bone defects showed a good stability of the marginal bone level
Covani U., 2006 [69]	DEH	Gen-Os^®^, Evolution	Similar results to those of immediate implants; success after prosthetic rehabilitation with no mobility, pain, suppuration, or peri-implant radiolucency (12-month follow-up)
Covani U., 2008 [70]	DEH	Gel 40, Evolution	Flap elevation provided higher regenerated bone at coronal level; immediate implants with or without flap elevation can be successful, even in the presence of bone defects
Covani U., 2009 [71]	DEH	mp3^®^, Evolution	Complete bone healing: no mobility, pain, suppuration, or peri-implant radiolucency at the second-stage surgery (6 months post-operation)
Slotte C., 2013 [72]	DEH	mp3^®^	Complete and enhanced bone regeneration with PCPB after 12 months: osteoconductive properties directly on the surface of the graft
Cassetta M., 2012 [73]	DEH	Gen-Os^®^, Putty	Stable long-term results for implants inserted in both groups (5-year follow-up)
Barone A., 2015 [74]	DEH	Apatos^®^, Evolution	Similar effectiveness and safety of immediate implant placement to delayed restoration; better healing times and costs
Barone A., 2016 [75]	DEH	mp3^®^, Evolution	Immediate implant placement and restoration showed predictable clinical outcomes with a very high success rate (7-year follow-up)
Ekstein J., 2016 [76]	DEH	Gen-Os^®^, Evolution	High crestal bone stability and limited marginal bone loss around conical connection tapered implants with platform switching; complete implant survival rate (14-month follow-up)
Covani U., 2014 [77]	DEH	Apatos^®^, Evolution	Positive final aesthetic results; minimal bone level changes; maintenance of the early increase in both midfacial tissue and the papillae (5-year prospective single-cohort study)
Figliuzzi M.M., 2015 [78]	DEH	mp3^®^, Evolution	No significant differences in the peri-implant bone reabsorption of post-extractive implants over 2 years
Zita Gomes R., 2017 [79]	DEH/LASL	mp3^®^, Evolution	Evaluating primary and secondary stability could lead to higher implant success

**Table 3:** Bone regeneration procedures with collagenated porcine xenografts: Crestal Access Sinus Lift (CASL), Lateral Access Sinus Lift (LASL) and Lateral Access Sinus Lift/Horizontal Augmentation (LASL/HOR).

Reference	Clinical Indication	Biomaterial	Results
Barone A., 2008 [80]	CASL	Gel 40, Evolution	Adequately performed, this technique showed no problems and clinical success predictability
Santagata M., 2010 [81]	CASL	Gel 40	This simplified treatment facilitated single tooth implant rehabilitation; immediate loading was easier thanks to improved bone density
Lopez M., 2016 [82]	CASL	Putty	Less traumatic and invasive surgery
Barone A., 2005 [83]	LASL	Gen-Os^®^, Evolution	No complications during surgical procedures; complete healing; no signs or symptoms of maxillary sinus disease (5-month after surgery)
Barone A., 2006 [84]	LASL	Gen-Os^®^, Evolution	More complications in smokers and with the use of onlay bone graft in conjunction with sinus augmentation
Orsini G., 2006 [85]	LASL	Apatos^®^, Evolution	New formed bone around particles; presence of the osteoid matrix in some areas; mainly compact bone present at the interface; no acute inflammatory infiltrate
Barone A., 2008 [86]	LASL	mp3^®^, Evolution	No significant differences in clinical parameters for piezosurgery and conventional instruments
Scarano A., 2009 [87]	LASL	Lamina^®^	Patient remained asymptomatic; no infections or inflammation from the implants migrated in the maxillary sinus (7-year after removal)
Scarano A., 2010 [88]	LASL	Apatos^®^, Evolution	Successfully results with porcine bone; rougher-surfaced implants were preferable; less peri-implant marginal bone resorption (5-year follow-up after loading)
Scarano A., 2011 [89]	LASL	Apatos^®^, Evolution	Cortical porcine bone was biocompatible, osteoconductive and did not interfere with the normal reparative bone processes (4- and 6-month after retrieval)
Hinze M., 2013 [90]	LASL	mp3^®^, Evolution, Lamina^®^	Minimized sinus infections; preserved integrity of the sinus membrane; regenerated bone around the zygomatic implants (6-month after implant placement)
Iezzi G., 2012 [91]	LASL	Apatos^®^	Success of all the biomaterials: newly formed bone and vessels thanks to the high microporosity; many grafted particles partially resorbed and substituted by newly formed bone (6-month follow-up)
Barone A., 2013 [92]	LASL	mp3^®^, Evolution	No significant increase in vital bone; reduced connective tissue proliferation and reabsorption of the graft; maybe blood supply can play a role in such a result (6-month follow-up)
Ramirez Fernandez M.P., 2013 [93]	LASL	mp3^®^, Evolution	Biocompatibility, bio-resorbability and osteoconductivity: newly formed bone on the xenografts; gradual diffusion of Ca^2+^ ions from the biomaterial into the newly forming bone at the interface (biomaterial reabsorption process)
Cassetta M., 2012 [94]	LASL	Gen-Os^®^, Evolution	Piezoelectric device could simplify sinus augmentation; better results in terms of sinus membrane perforations; no statistical differences in time for the antrostomy and sinus membrane elevation in respect to traditional instruments
Silvestri M., 2013 [95]	LASL	mp3^®^	After 6 months, PCPB resulted a valid and predictable alternative to DPBB
Traini T., 2015 [96]	LASL	Apatos^®^	None of the biomaterials seemed to be ideal: the regenerated bone had a D3 bone quality and covered almost one-third of the space filled by BSBs (6-month after healing)
Cassetta M., 2015 [97]	LASL	Gen-Os^®^	Porcine bone alone or with autologous bone showed biocompatibility and osteoconductivity; uneventful healing; comparable newly formed bone, marrow spaces and residual grafted material in the three groups (2-month follow-up)
Falisi G., 2013 [98]	LASL	mp3^®^	Functional and anatomic recovery of the maxillary antrum; immediate implant placement (diameter > 4 mm); reduced surgical times; no patient morbidity; local anesthesia (one-year follow-up)
Scarano A., 2014 [99]	LASL	Lamina^®^	Achieved bone formation and possible placement of implants without any grafting material: totally healed sinus’ wall; newly formed bone; wide osteocyte lacunae; large marrow spaces; newly formed vessels; no inflammation
Corbella S., 2016 [100]	LASL	Apatos^®^, mp3^®^, Gen-Os^®^	Use of AB to achieve the highest new bone formation; use of BB or a mixture of TCP and HP when donor site morbidity occurs
Lopez M., 2016 [101]	LASL	mp3^®^, Lamina^®^, Evolution	Good quality bone reformation: a new sinus floor filled with resorbable cortico-spongious bone paste; adequate vascularization of the graft; integration of the Lamina^®^
Iezzi G., 2017 [102]	LASL	Gen-Os^®^, Apatos^®^, mp3^®^	All the BSBs can be used successfully: biocompatibility; osteoconductivity; new bone surrounding many particles, crosslinked by newly formed bone trabeculae; gradual reabsorption and partial replacement by new bone; no adverse reactions
Esposito M., 2018 [103]	LASL	Sp-Block, mp3^®^, Evolution	Zygomatic implants showed more complications: significantly fewer prosthetic and implant failures and the need for functional loading; better rehabilitation of severely atrophic maxillae (4 months post-loading)
Forabosco A., 2018 [104]	LASL	Gen-Os^®^, Evolution	The use of PRF in combination with biomaterials or alone was effective and safe; low risk; satisfactory clinical results
Davò R., 2018 [105]	LASL	Sp-Block, mp3^®^, Evolution	Immediately loaded zygomatic implants showed more complications: significantly fewer prosthetic and implant failures and more time for functional loading; better rehabilitation of severely atrophic maxillae (one-year post-loading)
Bechara S., 2017 [106]	LASL	Gen-Os^®^, Evolution	Short implants (6 mm) showed no significant differences in the augmented bone results, faster treatment, and minor costs (3-year follow-up)
Chirilă L., 2016 [107]	LASL	Gen-Os^®^	Caution with all the procedures to not destroy the ostium, compromising maxillary sinus clearance; signs of infection disappeared within 5 to 7 days, and normal sinus function and drainage were restored
Noami S., 2014 [108]	LASL	mp3^®^, Evolution	Biocompatibility and osteoconductivity; most of the particles surrounded by newly formed bone; large osteocyte lacunae; some marrow spaces; new bone formation suggested by residual particles
Mehl C., 2016 [109]	LASL	mp3^®^	More time- and cost-effectiveness to allow comprehensive prosthetic restorations within a month, without using frequent and long treatments
Kawakami S., 2018 [110]	LASL	Gen-Os^®^, Evolution	Greater augmentation height when the antrostomy was placed more cranial; the sinus mucosa width regained the original dimensions (9 months after surgery)
Scarano A., 2018 [111]	LASL	Gen-Os^®^, Lamina^®^, Evolution	Successful mechanical support of sinus membranes; only bone tissue formation, not mixed with the graft; biocompatibility; not complete resorption after 6 months, but residual was bone integrated
Scarano A., 2018 [112]	LASL	Lamina^®^	Bone formation without using biomaterials; preserved space in sinus lifting, contributing to wound healing
Kawakami S., 2019 [113]	LASL	Gen-Os^®^, Evolution	The height of the antrostomy did not influence clinical and radiographic results in LASL
Hirota A., 2019 [114]	LASL	Gen-Os^®^, Evolution	No differences in clinical results on the dimensional changes of augmented maxillary sinus floor in perforated or not sinus mucosae (9 months after healing)
Tanaka K., 2019 [115]	LASL	Gen-Os^®^, Evolution	More mineralized bone and bone marrow and less amounts of soft tissue in the alveolar crest of the maxillary sinus (9 months after surgery)
Adiloglu S., 2019 [116]	LASL	Gen-Os^®^, mp3^®^, Evolution	Higher new bone formation with 100% collagenated bone mix; no differences in connective tissue formation and residual graft materials (6-month healing process)
Luongo R., 2020 [117]	LASL	Lamina^®^	The porcine cortical bone layer increased bone formation and implant stability; reduced healing time, cost, and biological complications (1- to 5-year follow-up)
Felice P., 2020 [118]	LASL	Sp-Block, mp3^®^, Evolution	Immediately loaded zygomatic implants reported fewer prosthesis failures, implant failures and functional loading time, but more complications over time (3 years post-loading)
Pagliani L., 2012 [119]	LASL/HOR	Gen-Os^®^, mp3^®^, Gel 40, Evolution, Lamina^®^	Porcine bone substitute and barrier membranes showed good clinical results; bone condensation and resorption properties (one-year post-loading)

**Table 4:** Bone regeneration procedures with collagenated porcine xenografts: Horizontal Augmentation (HOR), Vertical Augmentation (VER), Horizontal and Vertical Augmentation (HOR/VER) and Vertical Augmentation/Lateral Access Sinus Lift (VER/LASL).

Reference	Clinical Indication	Biomaterial	Results
Cassetta M., 2005 [120]	HOR	Gen-Os^®^	Autologous bone graft integration in 4 months; mastication promoted the transformation into lamellar bone; reliability demonstrated within the first year of function
Barone A., 2007 [121]	HOR	mp3^®^	Minimal bone loss after bone block graft and implant placement; successful treatment of severe maxillary atrophy with autogenous bone from the anterosuperior edge of iliac wing
Santagata M., 2011 [122]	HOR	Putty	MERE technique reduced morbidity and healing time; simple and reliable technique; ideal implant placement
Wachtel H., 2013 [123]	HOR	mp3^®^, Evolution, Lamina^®^	Sufficient bone augmentation without other augmentation procedures and quite complete reabsorption after 6 months
Rodriguez J., 2013 [124]	HOR	Dual-Block, Evolution	Longer implants placement without clinical limitations with minimal bone height; more implant stability; minimal neurological disturbance
Scarano A., 2011 [125]	HOR	Gen-Os^®^	Viable and safe procedure to avoid crestal resorption and fracture of buccal plate; increased horizontal bone in coronal area; no compromission of cortical vascularization; no dehiscence of the mucosa; no hypoesthesia from patients
Scarano A., 2015 [126]	HOR	Gen-Os^®^	This technique, in association with biomaterial, allowed horizontal bone gain, good biomaterial integration, no fractures of buccal plate, and implant success
Lopez M., 2015 [127]	HOR	mp3^®^, Lamina^®^, Evolution	Combination of resorbable cortical Lamina^®^ and other resorbable biomaterials of porcine origin led to good vascularization of the graft, newly formed bone, and complete integration of the Lamina^®^ without its removal
Lopez M., 2016 [128]	HOR	Putty, Lamina^®^	Combination of resorbable cortical Lamina^®^ and some graft materials which did not allow stability alone led to good vascularization and complete integration of the Lamina^®^
Amr A., 2017 [129]	HOR	Gen-Os^®^, Lamina^®^	Successful alternative to the autogenous onlay block bone graft because no significant differences were found
Del Corso M., 2013 [130]	HOR	Gen-Os^®^	Stable, functional, and aesthetic rehabilitation; no significant bone loss; same level of the peri-implant tissues around the implant collars; no dehiscence (4-year follow-up)
Checchi V., 2019 [131]	HOR	mp3^®^	Uneventful and complete healing of the screw stage; stable and osseointegrated implants; not completely good esthetic but functional results because the buccal profile was not thick enough (8 months after implant placement)
Rossi R., 2019 [132]	HOR	Gen-Os^®^, Lamina^®^	Insertion of standard diameter implants and subsequent restoration; the regenerated bone was not remodeled and/or resorbed after 4 years of occlusal loading
Scarano A., 2019 [133]	HOR	Lamina^®^	Uneventful healing; increased bone regeneration; decreased volume of residual cavity; prevention of tissue collapses within the defect and maintaining of structural integrity; no need for second surgery (up to 24 months after surgery)
Esposito M., 2020 [134]	HOR	mp3^®^, Lamina^®^	Less invasive, faster, and cheaper treatment; less associated morbidity; marginal bone loss around the implant (one year after loading)
Iezzi G., 2020 [135]	HOR	Gen-Os^®^	No significant difference in crestal bone loss; promising technique for rehabilitating patients with agenesis of the upper lateral incisors (24-month follow-up)
Scarano A., 2011 [7]	VER	Sp-Block, Gen-Os^®^, Evolution	The biomaterial rigidity allowed the elimination of miniscrews and miniplates, the simplification of the technique and the preservation of the space; no dehiscence of the mucosa at the marginal ridge; newly formed bone also in close contact with the biomaterial particles without any connective tissue or gaps
Felice P., 2012 [136]	VER	Sp-Block, Evolution	Elimination of chisels to complete bone osteotomy; reduction in postsurgical nerve disturbances and intraprocedure
Felice P., 2013 [11]	VER	Sp-Block, Evolution	Successful implant prosthetic rehabilitation; newly formed bone within the block; no foreign body reactions (4 months after surgery)
Barone A., 2017 [137]	VER	Sp-Block	No significant difference in volumetric bone remodeling and in the success of the graft between the two groups, though inlay technique showed higher success rate (4 months after surgery)
Felice P., 2017 [138]	VER	Sp-Block, Evolution	Heterologous bone blocks were preferred to autogenous ones because showed similar results, avoiding invasive harvesting surgeries (2- to 7-year follow-up)
Marconcini S., 2019 [139]	VER	mp3^®^, Sp-Block	Success of the implants in low residual vertical height conditions before placement; temporary postoperative paresthesia resolved in 2 months; important bone gain after 4 months; little peri-implant marginal bone loss (3 years after loading)
Bernardi S., 2018 [140]	VER	Sp-Block	Loss of implants and significant complications with longer implants (one-year follow-up)
Gheno E., 2014 [141]	HOR/VER	Sp-Block, C-Block, Evolution	Effective permeation of CGF through the bone scaffold; high bone regeneration; high clinical success rate (12-month follow-up)
Rossi R., 2016 [142]	HOR/VER	mp3^®^, Lamina^®^	Uneventful rehabilitation; the resorbable membrane was vascularized and integrated with soft and hard tissues; active remodeling of the graft and gradual substitution with new bone; no secondary surgery
Rossi R., 2017 [143]	HOR/VER	Gen-Os^®^, Lamina^®^	Good and predictable results; its placement and the added particulate bone graft provided blood supply, stability, good bone regeneration, and reabsorption; uneventful healing
Polis Yanes C., 2019 [144]	HOR/VER	Lamina^®^, Apatos^®^	Resorbable heterologous cortical Lamina^®^ showed better outcomes: new bone formation after 4 weeks from the GBR, less morbidity, and successful outcomes
Rossi R., 2019 [145]	HOR/VER	Gen-Os^®^, Lamina^®^	Successful restoration of complex cases; no morbidity; reabsorption of the Lamina^®^; good balance between the soft tissue and the restorations
Rossi R., 2020 [146]	HOR/VER	Gen-Os^®^, Lamina^®^	Reliable, manageable, and versatile material; successful outcomes in all the three procedures, but better results when Lamina^®^ was combined with xenogenic bone of similar origin
Esposito M., 2012 [147]	VER/LASL	Sp-Block, Gen-Os^®^, Evolution	6 × 4 mm implants showed slightly better results, especially in posterior mandibles bone augmentation: faster, cheaper, and more uneventful treatment (5-month follow-up)
Felice P., 2012 [148]	VER/LASL	Sp-Block, mp3^®^, Evolution	5 × 5 mm implants with a novel nanostructured calcium incorporated titanium surface showed similar results in posterior mandibles bone augmentation: faster, cheaper, and more uneventful treatment (4-month follow-up)
Pistilli R., 2013 [149]	VER/LASL	Sp-Block, mp3^®^, Evolution	5 × 5 mm implants with a novel nanostructured calcium incorporated titanium surface showed similar results, especially in posterior mandibles bone augmentation: faster, cheaper, and more uneventful treatment (one-year follow-up)
Esposito M., 2016 [150]	VER/LASL	Sp-Block, Gen-Os^®^, Evolution	4-mm length implants showed slightly better results, especially in posterior mandibles bone augmentation: faster, cheaper, and more uneventful treatment, despite fewer complications (4-month follow-up)
Bolle C., 2018 [151]	VER/LASL	Sp-Block, Gen-Os^®^, Evolution	4-mm length implants showed slightly better results, especially in posterior mandibles bone augmentation: faster, cheaper, and more uneventful treatment, despite fewer complications (one-year follow-up)
Gastaldi G., 2018 [152]	VER/LASL	Sp-Block, mp3^®^, Evolution	5 × 5 mm implants with a novel nanostructured calcium incorporated titanium surface showed similar results in posterior mandibles bone augmentation: faster, cheaper, and more uneventful treatment (3-year follow-up)
Pistilli R., 2013 [153]	VER/LASL	Sp-Block, Gen-Os^®^, Evolution	6 × 4 mm implants showed slightly better results, especially in posterior mandibles bone augmentation: faster, cheaper, and more uneventful treatment (one-year follow-up)
Felice P., 2018 [154]	VER/LASL	Sp-Block, Gen-Os^®^, Evolution	6 × 4 mm implants showed slightly better results, especially in posterior mandibles bone augmentation: faster, cheaper, and more uneventful treatment (3-year follow-up)
Esposito M., 2019 [155]	VER/LASL	Sp-Block, mp3^®^, Evolution	5 × 5 mm implants with a novel nanostructured calcium incorporated titanium surface showed similar results in posterior mandibles bone augmentation: faster, cheaper, and more uneventful treatment (5-year follow-up)
Felice P., 2019 [156]	VER/LASL	Sp-Block, Gen-Os^®^, Evolution	6 × 4 mm implants showed similar results, especially in posterior mandibles bone augmentation: faster, cheaper, and more uneventful treatment (5-year follow-up)
Esposito M., 2020 [157]	VER/LASL	Sp-Block, Gen-Os^®^, Evolution	4 mm long implants showed slightly better results, especially in posterior mandibles bone augmentation: faster, cheaper, and more uneventful treatment, despite fewer complications (3-year follow-up)

**Table 5:** Bone regeneration procedures with porcine xenografts: Maxillofacial (MAX).

Reference	Clinical Indication	Biomaterial	Results
Rinna C., 2005 [158]	MAX	Lamina^®^	Excellent results: avoiding autologous implants and greater morbidity; complete integration; regeneration of wide fractures without the use of metal mesh support; fewer costs (from 1 to 8-year follow-up)
Grenga P., 2009 [159]	MAX	Lamina^®^	Hess area ratio >85% had no postoperative diplopia; Hess area ratio <65% had postoperative diplopia; Hess area ratio between 65% and 85% had variable surgical outcomes, but most patients had no problematic diplopia (4 months post-surgery)
Rinna C., 2009 [160]	MAX	Lamina^®^	Excellent results: biocompatibility; adaptability; no damage to the orbital soft tissues during application; restoration of wide defects
Ozel B., 2015 [161]	MAX	Lamina^®^	Good results: biocompatibility; plasticity; no morbidity; no restoration of near-total or wide defects (1, 3, 6, and 12-month follow-up)
Cascone P., 2018 [162]	MAX	Lamina^®^	Valid results: 50% more incisal opening after the procedure; 31.8% less excursive movement to the right and 22% more to the left
Senese O., 2018 [163]	MAX	Lamina^®^	Transconjunctival approach is the best surgical technique with high patient satisfaction

**Table 6:** Bone regeneration procedures with collagenated porcine xenografts: Periodontal Regeneration (PER) and Soft Tissue Augmentation (TIS).

Reference	Clinical Indication	Biomaterial	Results
Del Corso M., 2008 [164]	PER	Gen-Os^®^	As a membrane: protection of the surgical site; accelerated wound healing of the soft tissues; reduced morbidity. With graft materials: attraction of mesenchymal cells and vessels; osteogenic effect; immune action
Cardaropoli D., 2009 [165]	PER	Gel 40, Evolution	85% initial ridge dimensions preservation; correct implant placement; newly formed bone with 25% residual graft particles (4 months post-extraction)
Fickl S., 2013 [166]	PER	Derma	Possible use to replace autologous material; complete root coverage only in 42.86% of the defects (6 and 12 months post-surgery)
Esposito M., 2015 [167]	PER	Gen-Os^®^, Evolution	Significant better results than open flap debridement in PAL gain, PPD reduction, and RAD gain in defects deeper than 3 mm
Attia A., 2017 [168]	PER	Gen-Os^®^	Significant clinical improvements of PI, GI, PD, and CAL: improved bone density and reduction in defect depth (6 and 12 months after surgery)
Aslan S., 2017 [169]	PER	Gen-Os^®^	Complete and uneventful wound healing with excellent clinical results: limited wound failure in the early phase; stabilization of blood clot in deep intra-bone defects, avoiding the exposure of regenerative biomaterials (one-year follow-up)
Aslan S., 2017 [170]	PER	Gen-Os^®^	Uneventful and complete wound healing of the interdental papilla (8-month follow-up)
Fischer K., 2014 [171]	TIS	Derma	Successful replacement of autologous grafts: less morbidity; less chair time; complete and uneventful wound healing and augmented ridge contour with ADM; successful gain of keratinized mucosa with CM
Matoh U., 2019 [172]	TIS	Derma	CM is a valid alternative to CTG: complete correction in 7/10 of sites and 85% +/− 24% of root coverage (12 months after treatment)
Fischer K., 2019 [173]	TIS	Derma	Significant soft tissue augmentation during all the follow-up period, despite graft shrinkage in the first 6 months; uneventful healing (6- and 24-month follow-up)
Verardi S., 2020 [174]	TIS	Derma	Significant thicker peri-implant soft tissues and more vertical gain with the porcine dermal matrix (6 months after placement)
Baldi N., 2020 [175]	TIS	Derma	Autologous connective tissue graft provided significant facial soft tissue gain and width augmentation of keratinized mucosa; uneventful healing (6-month follow-up)

**Table 7:** Bone regeneration procedures with collagenated porcine xenografts: Laboratory Tests (*in vitro* studies) (LAB), Laboratory Tests/Experimental Studies (LAB/EXP) and Laboratory Tests/Lateral Access Sinus Lift (LAB/LASL).

Reference	Clinical Indication	Biomaterial	Results
Trubiani O., 2007 [176]	LAB	Apatos^®^	At 30 days, PDL-MSCs were completely integrated into the 3D bio-scaffold; the biomaterial perfectly mimed the human bone and was osteoconductive
Figueiredo M., 2010 [20]	LAB	Gen-Os^®^	The biomaterials had different particle sizes, shapes, surface areas, organic material content, and total porosity (mainly submicron pores); Biocoral^®^ density values were similar to those of hydroxyapatite, while the values of the collagenated samples were lower; most of the samples were hydroxyapatite based
Brunelli G., 2011 [21]	LAB	Apatos^®^	Up-regulation of SPP1 and ALPL in ADSCs and hOBs and of COL1A1 in hOBs: active resorption of the biomaterial by human osteoclasts; osteoinductive properties; matrix synthesis and deposition in hOBs in the late differentiation (15 days of treatment)
Kolmas J., 2012 [177]	LAB	Gen-Os^®^, Apatos^®^	The biomaterials were mainly constituted by nanocrystalline apatite mineral, organic collagenous matrix, and water; crystal sizes and specific surfaces areas were similar tothose in bone mineral
Manescu A., 2016 [178]	LAB	Dual-Block	New mineralized bone formation from the second week of culture in basal and differentiating media, but more in the trabecular portion and in differentiating media
Rombouts C., 2016 [26]	LAB	Gen-Os^®^	Both Gen-Os^®^ (of equine and porcine origin) grafting materials showed a significant increase in VEGF secretion by PDLCs, endothelial cell proliferation, and angiogenesis
Barone A., 2014 [179]	LAB	Lamina^®^	Osteogenic phenotype induction in hMSCs grown on titanium discs but not on xenogenic bone; up-regulation of DLX5 and down-regulation of RUNX1 in cells cultured on titanium; up-regulation of DLX5, CTNNB1, and RUNX1, and SP7 down-regulation in OICs
De Marco P., 2017 [180]	LAB	Derma	Coated membranes did not release GO or induce inflammation, and were biocompatible; GO changed stiffness and membrane-AFM tip adhesion, increased the roughness and the total surface exposed to the cells
Radunovic M., 2017 [181]	LAB	Derma	Improved proliferation and differentiation of DPSCs, higher compatibility, higher expression of BMP2 and RUNX2, and lower PGE2, COX2, and TNFα levels on GO coated membranes at 14 and 28 days
Canullo L., 2018 [182]	LAB	Sp-Block, Lamina^®^	Increase in murine osteoblasts adhesion and protein adsorption in all grafted materials
Brunelli G., 2012 [183]	LAB	Apatos^®^	Induction of osteoblast differentiation in DPSCs, increasing FOSL1, RUNX2, and SPP1 and decreasing ENG; involvement in bone resorption
Mazzoni S., 2017 [184]	LAB	Dual-Block	Guided osteogenic differentiation of hPDLSCs in xeno-free cultures, showing an acceleration of the process of mineralization
Lauritano D., 2012 [185]	LAB	Apatos^®^	Biocompatibility; promotion of osteoblast differentiation and bone regeneration: up-regulation of FOSL1, SPP1, SP7, and ALPL, down-regulation of ENG, COL1A1, and COL3A1
Maté Sanchez de Val J., 2018 [186]	LAB	Gen-Os^®^	Different microstructure, similar high porosity (intra and interparticle) and crystallinity between synthetic and organic materials; higher density for the synthetic materials
Genova T., 2019 [187]	LAB	Sp-Block, Lamina^®^	No significant differences in degrees of contamination of bone grafts, providing the required sterility of the surface
Di Carlo R., 2019 [188]	LAB	Lamina^®^	Increase in calcium phosphate deposition, DPSCs proliferation, and roughness of Lamina^®^, reduction in toxicity, preservation of DPSCs membrane integrity
Caballé Serrano J., 2019 [189]	LAB	Lamina^®^, Evolution	Increase in hydration in porcine-derived barrier membranes and wettability in rough surfaces; higher stiffness in bone Lamina^®^
Ambrozewicz E., 2019 [190]	LAB	Gen-Os^®^, Apatos^®^	Vitamins D3 and K could protect osteoblasts from redox imbalance and lipid peroxidation, support cell growth, increasing DNA biosynthesis
Jeanneau C., 2020 [23]	LAB	Gen-Os^®^	Gen-Os^®^ material better increased C5a secretion and MSCs proliferation and recruitment toward injured PDLCs, also leading to bone regeneration
Canullo L., 2020 [191]	LAB	Lamina^®^, Sp-Block	PAT increased the early stage osteoconductivity and osseointegration of the bone grafting materials: improved osteoblast adhesion without affecting macrophage viability
Toledano M., 2020 [192]	LAB	Derma, Evolution	Derma was the most resistant to all degradation techniques; the most aggressive test was the bacterial collagenase solution (complete degradation of all membranes by 21 d)
Ettorre V., 2016 [193]	LAB/EXP	Apatos^®^	The homogeneous GO-coated PB granules were more resistant to fracture load, biocompatible did not trigger inflammatory responses in an animal study, and lost small GO particles
Mijiritsky E., 2017 [194]	LAB/EXP	Gen-Os^®^	The controlled release of bioactive growth factors from bone granules promoted bone regeneration *in vivo* and the increase in VEGF and bFGF markers *in vitro*
Diomede F., 2018 [195]	LAB/EXP	Evolution	CM + EVO membranes + hPDLSCs up-regulated COL5A1, COL16A1, and TGF β1 and down-regulated 26 genes involved in bone regeneration *in vitro* and showed a better osteogenic ability in calvaria repair *in vivo*
Diomede F., 2016 [196]	LAB/EXP	Dual-Block	DB showed biocompatibility, osteoinductive and osteoconductive properties *in vitro* and a precocious osteointegration and vascularization in mouse calvaria
Diomede F., 2018 [197]	LAB/EXP	Evolution	EVO + PEI-EVs + hPDLSCs showed biocompatibility and an osteogenic potential *in vitro* and *in vivo* for the treatment of calvarium and ossification trauma defects
Bergmann M., 2020 [198]	LAB/EXP	Gen-Os^®^	Complement components secreted by cultured pulp fibroblasts eliminate bacteria and support the early steps of dental tissue regeneration, and those secreted by cultured PLC induced BMMSC recruitment
Fernandez M., 2017 [199]	LAB/LASL	mp3^®^, Evolution	Typical HA structure with intraparticle pores; significant porosity, crystallinity, and calcium/phosphate differences; excellent biocompatibility and similarity to natural bone; greater osteoconductivity, but fewer resorption properties for sintered HA xenografts
Fernandez M., 2017 [200]	LAB/LASL	mp3^®^	Significant decrease in Ca^2+^/P ratio, high porosity, low crystallinity, low density, large surface area, poor stability, and a high resorption rate in the residual biomaterial of the low-temperature sintered group (6 months after surgery)

**Table 8:** Bone regeneration procedures with collagenated porcine xenografts: Experimental Studies (EXP).

Reference	Clinical Indication	Biomaterial	Results
Nannmark U., 2008 [25]	EXP	mp3^®^, Gen-Os^®^, Evolution	Mixing collagen gel did not affect bone tissue responses: direct bone formation on the particles, increased bone area within 8 weeks, PCPB resorption with osteons formation, and PCPB area decrease within 8 weeks for both groups
Nannmark U., 2010 [201]	EXP	Putty, Gel 40	No differences in bone tissue response after changing collagen/CPB ratios: high bone formation rate and initial resorption after 8 weeks
Figueiredo A., 2013 [202]	EXP	Gen-Os^®^	OsteoBiol^®^ granules were larger, irregular, sharp-edge tips and for that triggered less inflammatory response; a bone-like structure and composition
Fickl S., 2015 [203]	EXP	Derma	No significant differences in foreign body reaction, tissue thickness, and height between the two groups (4-month follow-up)
Fischer K., 2015 [204]	EXP	Gen-Os^®^	Delayed healing of the extraction socket; reduced post-extraction horizontal bone width; obstruction of the resorption of the porcine bone substitute by pamidronate
Cakir M., 2015 [205]	EXP	Apatos^®^, Evolution	High biocompatibility of the materials; accelerated bone healing, bone formation, and graft degeneration with ABS alone or combined with CHBG; osteoconductive properties of CHBG (1, 4, and 8 weeks after surgery)
Scarano A., 2016 [206]	EXP	Gen-Os^®^, mp3^®^, Sp-Block, Evolution	Faster and higher bone regeneration and higher biocompatibility with scaffold of particulate porcine bone mix and porcine corticocancellous collagenated pre-hydrated bone mix (4 months after surgery)
Scarano A., 2017 [207]	EXP	C-Block	Higher bone regeneration with BDPSCs-BPB scaffolds (3 months after surgery)
Iida T., 2017 [208]	EXP	Gen-Os^®^, Evolution	No significant morphometric difference after placement of a collagen membrane subjacent the sinus mucosa; no complete resorption of the collagen membrane after 8 weeks (2, 4, and 8 weeks after surgery)
Omori Y., 2018 [209]	EXP	Gen-Os^®^, Evolution	No difference in bone augmentation area and bone density in respect to the coverage of the antrostomy fixed with a cyanoacrylate; incorporation of the repositioned bone plate after 8 weeks; residual defects in both groups (2, 4 and 8 weeks after surgery)
Develioglu H., 2018 [210]	EXP	Gen-Os^®^, Gel 40	Higher bone formation and osteoconductivity in both test groups, mostly in Gel 40 group, despite mild inflammation and graft resorption
Nemtoi A., 2017 [211]	EXP	mp3^®^, Lamina	Biocompatibility and osteoconductive capacity, mild inflammation in the early phase, partial and sequential graft resorption with collagenated porcine bone grafts in both healthy subjects and those with controlled diabetes; similar results in diabetic patients treated with Insulin and strontium ranelate
Iida T., 2018 [212]	EXP	Gen-Os^®^, Evolution	Increased bone formation, mostly close to the sinus bone walls in histological analyses; more bone formation in the middle regions with micro-CT analyses, especially after 2 weeks (2, 4, and 8 weeks after surgery)
Diker N., 2018 [213]	EXP	Gen-Os^®^	Xenogenic graft augmentation combined with EPO treatment significantly increased bone formation and vascularization; EPO helped the regenerative process of critical size bone defects
Kizilaslan S., 2020 [214]	EXP	Gen-Os^®^	Higher bone healing rate with xenogenous graft combined with CGF both in healthy and diabetic patients (6 weeks after surgery)
Favero G., 2020 [215]	EXP	Gen-Os^®^, Evolution	Small increase in bone formation after placement of autogenous bone and significant increase in the subjacent close-to-window region (1 and 8 weeks after surgery)
Giuliani A., 2020 [216]	EXP	Evolution	The use of CM, EVs, and PEI-EVs often fastened bone remodeling kinetics and the mineralization process (COL-hPDLSCs-PEI-EVs and PLA-hGMSCs-CM); better osteogenic capacity with CM (6 weeks after grating)
Fischer K., 2020 [217]	EXP	Gen-Os^®^, Derma, Lamina^®^	Uneventful healing; Lamina^®^ stability allowed bone formation and the inhibition of soft tissue invasion and was degradated; Gen-Os^®^ allowed bone regeneration and was resorbed (4 months after surgery)
Aragoneses J., 2021 [218]	EXP	Derma	Thicker keratinized tissue with MD: higher values at 15 d, decreased values at 45 d, and similar to control at 90 d (15, 45, and 90 d after surgery)

## Data Availability

All the data supporting reported results regarding publications cited in this review are available contacting the corresponding author upon request.
